# Therapeutic Strategies to Ameliorate Neuronal Damage in Epilepsy by Regulating Oxidative Stress, Mitochondrial Dysfunction, and Neuroinflammation

**DOI:** 10.3390/brainsci13050784

**Published:** 2023-05-11

**Authors:** Sahithi Madireddy, Samskruthi Madireddy

**Affiliations:** 1 Department of Biology, Massachusetts Institute of Technology, Cambridge, MA 02139, USA; 2 Department of Neuroscience, Johns Hopkins University, Baltimore, MD 21218, USA

**Keywords:** epilepsy, oxidative stress, mitochondrial dysfunction, inflammation, antioxidants, antiepileptic drugs, antiseizure medications, neuromodulation, keto diet, nutrients

## Abstract

Epilepsy is a central nervous system disorder involving spontaneous and recurring seizures that affects 50 million individuals globally. Because approximately one-third of patients with epilepsy do not respond to drug therapy, the development of new therapeutic strategies against epilepsy could be beneficial. Oxidative stress and mitochondrial dysfunction are frequently observed in epilepsy. Additionally, neuroinflammation is increasingly understood to contribute to the pathogenesis of epilepsy. Mitochondrial dysfunction is also recognized for its contributions to neuronal excitability and apoptosis, which can lead to neuronal loss in epilepsy. This review focuses on the roles of oxidative damage, mitochondrial dysfunction, NAPDH oxidase, the blood–brain barrier, excitotoxicity, and neuroinflammation in the development of epilepsy. We also review the therapies used to treat epilepsy and prevent seizures, including anti-seizure medications, anti-epileptic drugs, anti-inflammatory therapies, and antioxidant therapies. In addition, we review the use of neuromodulation and surgery in the treatment of epilepsy. Finally, we present the role of dietary and nutritional strategies in the management of epilepsy, including the ketogenic diet and the intake of vitamins, polyphenols, and flavonoids. By reviewing available interventions and research on the pathophysiology of epilepsy, this review points to areas of further development for therapies that can manage epilepsy.

## 1. Introduction

Epilepsy is a chronic neurological disorder characterized by unprovoked and repeated seizures that occurs in millions of people globally [1,2,3,4,5,6,7,8,9]. Epilepsy has serious cognitive, social, psychological, and economic consequences [10,11]. Epileptic seizures can seriously lower the quality of life when uncontrolled. Epilepsy arises from an increased frequency and synchrony of neuronal firing and an imbalance of excitatory neurotransmitters over inhibitory neurotransmitters [12]. Focal-onset seizures most frequently occur in the temporal lobe, making temporal lobe epilepsy (TLE) the most common form of epilepsy, which is also marked by impaired learning and memory [13,14,15]. Recurring and unpredictable partial complex seizures occur in TLE, which comprises 60% of all cases of epilepsy [16]. Status epilepticus (SE), another form of epileptic seizure defined by convulsive seizure activity lasting more than 5 min, results in high morbidity and mortality [17,18].

Epileptic seizures can lead to the death of neurons, which in turn promotes epileptogenesis and the occurrence of seizures [19,20,21]. The proposed mechanisms of epileptogenesis involve alterations in synapses, neurotransmitters, receptors, oxidative stress, mitochondrial dysfunction, cytokine signaling, and apoptosis [22,23]. A growing body of evidence links the development of epilepsy to the presence of oxidative stress and overproduction of reactive oxygen species (ROS) [24,25,26]. Prior to the onset of seizures, oxidative stress induces neurological changes, including inflammation, neurodegeneration, and a lowered seizure threshold, resulting in epileptogenesis [24,27]. By altering Ca^2+^ homeostasis, oxidative stress hastens seizure onset, neurodegeneration, and neuronal excitability [28,29]. Experimental evidence indicates that inflammation in the brain is also associated with epilepsy [30,31]. Neuroinflammation has been observed in both animal models of epilepsy and patients with epilepsy [32,33,34]. Chronic neuroinflammation causes peripheral immune cells, astrocytes, microglia, and endothelial cells in the blood–brain barrier (BBB) to produce inflammatory molecules [35].

The discovery of novel anti-epileptic therapies necessitates understanding contributors to the onset of epilepsy to identify therapeutic targets [36]. This review focuses on the roles of oxidative stress, mitochondrial dysfunction, inflammation, NADPH oxidase (NOX), neuronal excitotoxicity, and BBB dysfunction in the pathogenesis of epilepsy. Anti-inflammatory medications, antioxidants, anti-epileptic drugs (AEDs), and anti-seizure medications (ASMs) are used to treat epilepsy and manage its progression [37,38,39]. Current knowledge on the treatment of epilepsy with AEDs and ASMs is presented in this review, along with information on the potential nutritional and pharmacological regulation of antioxidant capacity and inflammation in patients with epilepsy. We discuss the use of antioxidants, ASMs, and AEDs, including acetyl-l-carnitine (ALC), melatonin, N-acetylcysteine (NAC), baicalein, coenzyme Q10 (CoQ10), astaxanthin, curcumin, valproic acid, levetiracetam, cannabidiol (CBD), brivaracetam, and ursolic acid. Although a wide number of medications against epilepsy are available, approximately one-third of patients do not respond to currently available pharmaceuticals [40,41]. In addition, we discuss the use of non-pharmacological interventions such as neuromodulation, including vagus nerve stimulation (VNS), and surgery to treat epilepsy. We also present the role of diets, including the ketogenic diet, and nutrients, including vitamins, polyphenols, and flavonoids, in epilepsy treatment. A search was conducted in the PubMed/Medline database using appropriate keywords (epilepsy, oxidative stress, mitochondrial dysfunction, inflammation, antioxidants, antiepileptic drugs, antiseizure medications, neuromodulation, keto diet, and nutrients). This search revealed a rapid expansion of the literature on the role of oxidative stress, mitochondria dysfunction, and inflammation on the pathogenesis of epilepsy, as well as treatment for epilepsy. Around 1000 articles were finalized for this review, and of those, 358 articles were used in this manuscript.

## 2. Epilepsy and Oxidative Stress

Oxidative stress, which can contribute to the onset of diseases such as epilepsy, describes an imbalance between the generation and removal of ROS/reactive nitrogen species (RNS) [42,43,44]. Aerobically active organs are particularly susceptible to the generation of free radicals and ROS because of the premature leakage of electrons from the electron transport chain [45,46,47]. One product of the transfer of electrons to O_2_ is superoxide anions (O_2_^•−^). To counteract this, superoxide dismutase (SOD) converts O_2_^•−^ to H_2_O_2_, which is converted by glutathione peroxidase (GPX) and catalase (CAT) to water and oxygen [48,49,50,51,52,53]. At baseline, 1–5% of a cell’s oxygen consumption is used to generate ROS, but this rate can be elevated by altered mitochondrial homeostasis, such as that in the setting of Ca^2+^ overload [47]. Oxidative stress ultimately causes cellular damage through lipid, DNA, and protein oxidation [54,55,56,57,58,59,60,61,62]. Oxidative damage can occur, especially in the iron–sulfur clusters in complexes I and III of the electron transport chain [56]. Due to its high metabolic requirements, the brain actively conducts aerobic metabolism, which makes it uniquely vulnerable to oxidative stress [63]. In addition, iron is abundant in the brain because of its necessity for neurological functioning, although its presence also increases the susceptibility to oxidative stress [64]. Seizures have been observed to induce ROS/RNS production, resulting in oxidative stress and subsequent cellular damage [65,66]. Inhibiting ROS production has been indicated to prevent the neuronal damage that accompanies epileptic seizures [67,68].

Clinical and experimental studies indicate that oxidative stress is both a cause and consequence of the progression of epilepsy [69,70]. Various models of epilepsy are associated with increases in the levels of oxidative stress biomarkers [71]. For example, in chemical convulsion models of epilepsy induced by the administration of pentylenetetrazol (PTZ), kainic acid (KA), or pilocarpine, the levels of F2-isoprostanes, which are markers of lipid peroxidation, were increased in brain areas, including hippocampal regions [71]. At the same time, the activities of antioxidant enzymes, including SOD, CAT, and GPx, were reduced [62,71]. Patients with TLE also displayed greater levels of peripheral blood markers of oxidative damage [72,73]. Patients with SE exhibited decreased plasma activities of SOD, CAT, and glutathione (GSH) and decreased serum total antioxidant capacity [74].

Epileptic seizures induce oxidative stress, which can cause further neuronal damage and lead to the development of subsequent seizures in a chain reaction [75]. Acute seizures result in excess ROS formation through increased mitochondrial dysfunction and increased NOX activity [76,77,78]. Additionally, glutamate receptor activation and excitotoxicity, which are two mechanisms of brain injury in epilepsy, contribute to oxidative stress [79]. The persistent neuronal firing that accompanies epilepsy can lead to the formation of free radicals, which can leak from the electron transport chain and react with oxygen to cause oxidative stress [43]. Consistent with this, persistent epileptic seizures have been found to result in nucleic acid, lipid, and protein oxidation, leading to cellular damage [70].

**Figure 1:**
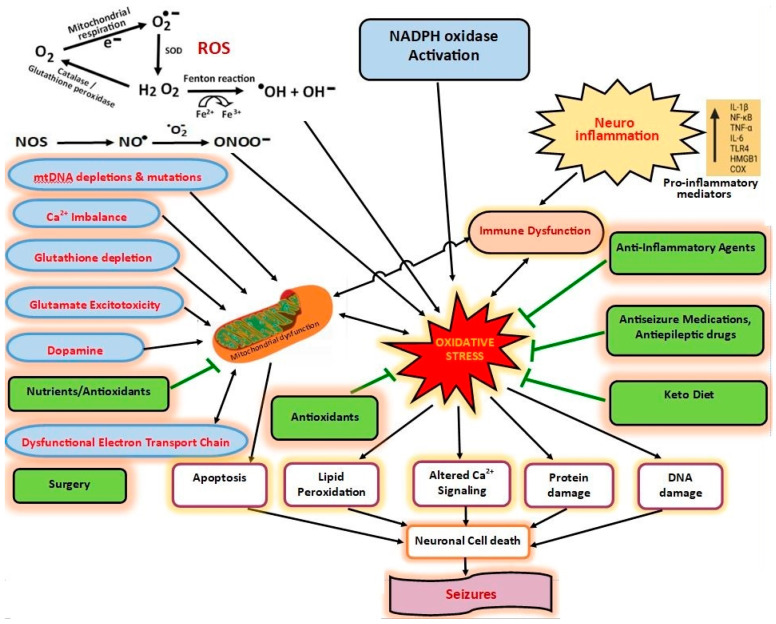
Potential interactions between seizures, oxidative stress, mitochondrial dysfunction, neuro-inflammation, antioxidants, ASMs, AEDs, anti-inflammatory agents, nutrients, and keto diet.

Both animal models and genetic studies support that oxidative and nitrosative stress induced by recurrent seizures leads to neuronal death [18,80]. The development of epilepsy is associated with neuronal loss through apoptosis [69]. For instance, patients with epilepsy exhibit a progressive decline in hippocampal size, resulting in additional severe seizures and cognitive deficits [69]. A single instance of SE in animal models produces long-standing changes within mitochondria, including mitochondrial DNA (mtDNA) damage and excess hydrogen peroxide production in the inner mitochondrial membrane [70]. One mechanism by which oxidative stress has a causative role in epilepsy is by inducing neuronal hyperexcitability, which is a key feature of epilepsy [70]. Moreover, mtDNA mutations that cause metabolic dysfunction in neurons can give rise to genetic epilepsy, further indicating that oxidative stress can contribute to epileptogenesis [68,70]. Intracellular damage induced by ROS is frequently observed in epileptic brain samples following surgical resection, which is consistent with the potential causative role of oxidative stress in epileptic processes, including neurodegeneration and neuronal hyperexcitability [73,79]. Figure 1 illustrates the potential interactions between seizures, oxidative stress, mitochondrial dysfunction, neuro-inflammation, antioxidants, antiseizure medications, antiepileptic drugs, anti-inflammatory agents, nutrients, and the keto diet.

## 3. Epilepsy and Mitochondrial Dysfunction

Mitochondria are organelles that function in energy generation, which is crucial for neuronal activity [81]. The brain’s high energy requirements make it dependent on mitochondria, which are involved in neurotransmitter synthesis, Ca^2+^ sequestration, redox signaling, and cell death [81]. Mitochondria are essential in ATP synthesis through oxidative phosphorylation, as well as fatty acid oxidation, glutamate and urea metabolism, and antioxidant activity regulation [19,82,83]. Mitochondrial dysfunction leads to altered neurotransmission and neuronal excitability [84,85]. Because mtDNA is close to the site of ATP synthesis, its 37 genes are especially susceptible to oxidative damage [86]. ROS can leak from the mitochondrial electron transport chain, thereby contributing to oxidative damage in the mitochondria, mitochondrial dysfunction, and subsequent tissue injury [82,87,88].

Several forms of epilepsy are associated with impaired mitochondrial function and increased ROS generation [77,89,90]. Moreover, mitochondrial dysfunction has been proposed as one cause of seizure occurrence in epilepsy [79,91]. This is supported by epileptic seizures being a symptom of genetic mitochondrial diseases involving mtDNA and nuclear DNA mutations [92]. Specifically, mtDNA damage has been suggested to contribute to the development of epilepsy [93]. mtDNA oxidative damage and increased mitochondrial hydrogen peroxide were observed in a KA-induced TLE model [94]. Studies in rats treated with KA and pilocarpine also indicated that mitochondrial oxidative stress results in oxidative damage to DNA during epileptogenesis [95]. Similarly, animal models of epilepsy induced by homocysteic acid were observed to have mitochondrial dysfunction [91].

One manner in which mtDNA damage from oxidation can cause epileptogenesis is through inhibiting mitochondrial base excision repair, leading to neuronal apoptosis [94,96]. Additionally, ROS can promote the opening of the mitochondrial permeability transition pore (MPTP), which leads to an efflux of ions and mitochondrial molecules that ultimately cause cell death [73]. Decreases in neuronal ATP and increased mitochondrial Ca^2+^ levels have been observed during seizures [83]. An excess of mitochondrial Ca^2+^ can lead to the generation of ROS through xanthine oxidase activation and through other pathways, as well as to the production of RNS [97].

## 4. Lipid Peroxidation

Epileptic seizures can also cause oxidative damage to intracellular lipids [98,99]. Polyunsaturated fatty acids within phospholipid bilayers surrounding cells and organelles are particularly vulnerable to oxidation [98]. Similar to protein oxidation and mtDNA damage, the brain is also at risk of lipid peroxidation following seizures [98]. After seizures, Ca^2+^ can activate phospholipase A_2_, which releases arachidonic acid [98]. The metabolism of arachidonic acid can lead to the further formation of ROS. The peroxidation of arachidonic acid leads to the generation of F2-isoprostanes and isofurans through catalysis by free radicals [100]. After KA administration, seizures were observed to increase F2-isoprostane and isofuran levels in several hippocampal regions [101]. The appearance of additional lipid peroxidation markers, including 4-hydroxy-2-(E)-nonenal and malondialdehyde, indicated oxidative damage to lipids occurring within 4 h into an SE episode and up to 24 h afterward [102]. This suggests that lipid peroxidation is a consequence of seizure activity, and it may be a component of epileptogenesis.

## 5. Epilepsy and Inflammation

Inflammatory molecules can bind to surface receptors on neurons and other brain cells to activate signaling pathways [103]. Accumulating evidence suggests that inflammation contributes to seizure onset and epileptogenesis [104,105,106]. Signaling downstream of inflammation can lead to neuronal damage, which contributes to the clinical manifestations of pathology [107,108]. Meanwhile, seizures can induce neuroinflammation, and repetitive seizures might result in chronic inflammation [109]. This can lead to a disruption of the brain’s cytokine balance, further contributing to the progression of epilepsy. The production of inflammatory cytokines induces the generation of free radicals and alters glutamatergic synaptic transmission in a manner that promotes excitotoxicity [110,111]. In chronic epilepsy, long-term hyperexcitability and impaired synaptic transmission are observed in central nervous system (CNS) tissue following persistent inflammation [112,113]. In addition, neuroinflammation attributable to brain injury from repetitive seizures can lead to glial activation, which contributes to the occurrence of secondary seizures [31].

Several studies indicated that repetitive epileptic seizures are associated with increased levels of pro-inflammatory cytokines, including TNF-α, IL-1β, and IL-6; additionally, they are associated with increases in the protein expression of caspase-3, BAX, and BH3, which are involved in apoptosis and neurodegeneration [39,64,114,115]. After seizures, patients with epilepsy were observed to have elevated serum and cerebrospinal fluid TNF-α, IL-1β, IL-6, and IL-1 receptor antagonist levels [116,117]. The onset and spread of seizures were also found to induce rapid regional inflammatory responses in animal models of epilepsy [118]. Chronic neuroinflammation can contribute to epilepsy through mechanisms such as elevated TNF-α expression, promoting hyperexcitability and the activation of AP-1, which regulates apoptosis through pathways including JNK signaling [114,115]. This is consistent with apoptosis being a cause of neuronal death during the progression of epilepsy [119]. Cytokine production after seizures is observed in various cell types in areas of seizure onset, including glia, myeloid cells, and neurons [120,121]. The release of cytokines from microglia is suggested to support epileptogenesis by aggravating oxidative stress in the mitochondria [114,122].

Anti-inflammatory drugs hold promise for treating epilepsy, with favorable clinical evidence supporting inflammation suppression as a strategy for ameliorating the pathology of epilepsy [108,123]. Therefore, anti-inflammatory drugs may be beneficial in managing epilepsy, preventing seizure progression, and protecting against cognitive deficits [38,39].

## 6. Epilepsy and NOX

NOX is an enzyme complex that generates cellular ROS and promotes neurodegeneration, neurotoxicity, and memory deficits. Therefore, it has been suspected to be involved in epileptogenesis [24,124]. The NOX family includes seven isoforms (NOX1–5, DUOX1, DUOX2) that generate H_2_O_2_ by transferring an electron from NADPH to oxygen [125]. A mouse model of epilepsy induced by PTZ treatment displayed oxidative stress, altered neurotransmission, memory deficits, and anxiety-like and depression-like behavior, which were alleviated by NOX inhibition [42].

Accumulating evidence indicates that NOX is a mediator of epilepsy progression [10]. In animal models of epilepsy, NOX has been shown to be a key source of ROS during seizures and a contributor to neuronal death and neurodegeneration [24,77,126,127]. In particular, NOX2 is a major source of ROS generated in the presence of seizure activity [83]. NOX activation after PTZ treatment, along with mitochondrial damage, leads to ROS/RNS formation, decreased antioxidant enzyme levels, lipid peroxidation, elevated nitrite levels, and ultimately limbic neurodegeneration [128,129]. In addition, NOX2 elevation has been observed in neurons and glia of surgically resected sites where seizure activity originates in patients with refractory epilepsy. This further suggests that NOX2 activity is involved in epileptogenesis [130]. NOX2 activation has been observed in early epileptic seizures attributable to hyperactivated NMDA receptors [77,131,132].

Inhibition of NOX2 activity can suppress neuronal death caused by seizures in different models of epilepsy [77,133,134,135]. In one study investigating treatment with gp91ds-tat, a competitive inhibitor of NOX2, gp91ds-tat prevented cellular changes downstream of in vitro seizure-like activity, including Ca^2+^ oscillation, ROS formation, mitochondrial depolarization, and neuronal loss [24]. Additionally, gp91ds-tat treatment in a rat model 1 h after KA-induced SE led to reduced NOX2 expression and decreased cortical and hippocampal NOX activity [24]. Continuous intracerebroventricular injection of gp91ds-tat also decreased the occurrence of seizures in a rat model of epilepsy [24]. Overall, the anti-seizure activity of gp91ds-tat suggests that NOX2 can contribute to epileptogenic processes, including seizure development, oxidative stress, and ROS formation [24].

## 7. Epilepsy and Excitotoxicity

A fundamental feature of the pathogenesis of epilepsy is an imbalance between excitatory and inhibitory neurotransmission [136]. The levels of glutamate, an excitatory neurotransmitter, have been reported to be unusually elevated in both patients with epilepsy and animal models of epilepsy [10]. One consequence of excessive glutamatergic neurotransmission and glutamate receptor activation is oxidative stress, which leads to excitotoxicity, one form of neuronal apoptosis [62]. Glutamate promotes the activation of NMDA receptor-mediated Ca^2+^ influx into neurons [10]. Accumulated Ca^2+^ leads to neuronal depolarization, ROS formation through the arachidonic acid cascade, and eventual apoptosis [135,137]. ROS downstream of Ca^2+^ influx can further alter glutamate receptors, damage glutamate transporters, and contribute to oxidative stress by reducing GSH production [138,139]. This leads to a state of hyperexcitability and eventual neuronal death. Excessive ROS generation is a prerequisite for neuronal excitotoxicity, which is a well-characterized feature of epilepsy [10].

Meanwhile, GABA is the major inhibitory neurotransmitter of the CNS [136]. GABA can bind to GABA_A_ receptors, which are heteromeric ligand-gated Cl^−^ channels. Therefore, GABA stimulates these receptors to permit an influx of Cl^−^ [136]. These ions decrease depolarization in neurons to dampen the effects of excitatory signals [140]. When the inhibitory input from GABA binding to GABA_A_ receptors is inhibited, neurons undergo hyperexcitability and apoptosis [141]. ROS modulates both synaptic and extrasynaptic inhibition by GABA at hippocampal and cerebellar GABA_A_ receptors [142,143]. Because of GABA’s role in epilepsy, GABA receptors are targets of several anti-seizure drugs.

## 8. BBB Dysfunction

The BBB consists of endothelial cells that limit the transfer of molecules and pathogens between the bloodstream and brain tissue [144]. The BBB’s tight junctions protect the brain against infection and maintain homeostasis by strictly regulating the influx and efflux of substances [144]. Leakage of the BBB is proposed to be both a cause and consequence of epileptic seizures [145]. Glutamate signaling in seizures can increase the expression of matrix metalloproteinase, a tissue-remodeling enzyme that degrades extracellular matrix components. It can also cause reduced tight junction protein expression [146]. These two mechanisms contribute to BBB leakage triggered by seizures. Conversely, BBB leakage can also aggravate epilepsy [147]. Blood leakage through the BBB can increase the extracellular levels of glutamate and potassium, which increase neuron excitability and reduce the seizure threshold, increasing the likelihood of seizures [148]. The entry of albumin and other serum proteins also induces neuronal hyperexcitability and inflammation through cytokine production [147]. A disrupted BBB could also permit more leukocytes to enter the brain, potentially contributing to epileptogenic neuroinflammation [145]. Another way in which the BBB can affect the course of epilepsy is by blocking the entry of ASMs and AEDs and increasing their efflux from the brain, which can result in treatment-resistant epilepsy [145].

## 9. Epilepsy and Antioxidants (Antioxidant Therapies)

Antioxidants are molecules that counteract ROS, which, if uncontrolled, can lead to oxidative stress [149,150,151,152]. There are many substances with antioxidant properties, including vitamins A, C, and E; polyphenols; and GSH, the functions of which are aided by several antioxidant enzymes [40]. Antioxidants can balance ROS by a number of molecular mechanisms. For instance, they can restrict ROS generation either physically or by binding metal ions. Once ROS are generated, antioxidants function to neutralize ROS, chemically quench their activity, or otherwise catalyze their neutralization [153]. They can also disrupt radical chain reactions, scavenging ROS before they are able to cause cellular damage [153].

Due to their major neuroprotective role, antioxidant therapy has increasingly been considered a promising approach for treating diseases involving neurodegeneration [149,154]. Research in this area has suggested that antioxidants such as vitamin C, vitamin E, polyphenols, melatonin, lipoic acid, and NAC effectively limit oxidative-stress-associated neurodegeneration in drug-resistant epilepsy [99]. Therefore, antioxidant therapy aimed at decreasing oxidative stress can be helpful in alleviating seizures in patients with drug-resistant epilepsy [155]. Specifically, recent studies demonstrated that antioxidants protect cells from the neurotoxic effects of seizures [156,157]. For instance, vitamin E has been shown to effectively inhibit ferroptosis, one method of neuronal death, following epileptic seizures [10]. In another study, Alzoubi et al. investigated the effect of vitamin E supplementation on epileptic seizures by feeding rats with control, a high-fat diet (HFD), vitamin E, or vitamin E combined with an HFD over 6 weeks [149]. They found that although the HFD normally increased susceptibility to PTZ-induced seizures, this effect could be prevented by vitamin E supplementation, likely through its strengthening of the hippocampal antioxidant mechanism [149]. Although antioxidants have multiple forms and sources, medicinal plants have been increasingly studied as sources of natural antioxidants, including phenolic acids, carotenoids, and flavonoids, which exhibit particularly strong antioxidant properties [158].

### 9.1. Acetyl-l-carnitine

ALC is a modified amino acid that naturally occurs in the body and can cross the BBB, allowing it to exert neuroprotective effects by inhibiting oxidative stress and apoptosis, as well as glial activation and neuroinflammation [159,160]. Research has demonstrated that through these mechanisms, ALC can effectively attenuate SE. In 1 study using a KA model of TLE, rats treated with 100 mg/kg ALC showed reduced neuronal loss and seizure intensity and attenuated a higher incidence of SE [29].

### 9.2. Melatonin

Melatonin has been shown to have neuroprotective effects in human epilepsy and in various animal models [161,162,163]. For instance, prior studies demonstrated that melatonin reduced the incidence of iron-induced seizures and increased the initial seizure latency in pilocarpine- and penicillin-induced seizure models [164]. Some researchers reported the therapeutic effects of melatonin in the PTZ model, which potentially involved the regulation of GABA receptors and the inhibition of neuronal nitric oxide synthase activity to interfere with glutamatergic pathways. Similarly, studies using the KA model found that melatonin prevented the neurotoxic effects of seizures, including ROS production, mtDNA damage, lipid peroxidation, hippocampal cell loss, and decreased GSH and mitochondrial complex II activity [165,166,167]. It is worth noting that one study found no significant neuroprotective effects of melatonin in PKZ and KA models [168]. Overall, research suggests that melatonin is an effective component of strategies for treating epilepsy.

### 9.3. NAC

NAC is a precursor to GSH that is used clinically to prevent the oxidative stress-induced depletion of GSH [169,170]. NAC also counters oxidative stress through its own antioxidant properties, including donating sulfhydryl groups to directly scavenge free radicals [171]. A study in which NAC was administered at 500 mg/kg twice daily along with 5 mg/kg sulforaphane daily in a rat model of SE observed a substantial neuroprotective effect [171]. NAC and sulforaphane treatment led to a 70% decrease in seizure frequency, a 30% increase in the time to the onset of epileptic seizures, and the amelioration of cognitive impairments accompanying epileptogenesis [172]. In another study of a fluid percussion injury model of epilepsy in rats, chronic NAC treatment reduced the seizure threshold to a level comparable to that of PTZ-induced seizures as opposed to what would be expected following brain injury [173]. Additionally, patients with Unverricht–Lundborg disease, a form of genetic epilepsy, tolerated NAC well, and they had a reduced seizure burden after several months of treatment [174].

### 9.4. Baicalein

Baicalein is another compound with bioactive properties relevant to protection against neurodegeneration in various brain disorders [175,176]. One study examined the effects of baicalein injections in rats with spontaneous recurrent seizures. Although there was no apparent reduction in the frequency of these spontaneous recurrent seizures, rats treated with baicalein showed better cognition and reduced mossy fiber sprouting and hippocampal cell loss [1]. These results were attributed to baicalein’s antioxidant and anti-inflammatory properties, the regulation of synapse-associated proteins, and the recovery of glucocorticoid pathway function, all of which were observed in this study [1]. These findings indicate that baicalein is a beneficial adjuvant therapy in epilepsy.

### 9.5. CoQ10

CoQ10 is a potent endogenous antioxidant that protects against ROS generation and oxidative damage [177,178]. CoQ10 both directly scavenges free radicals and indirectly regenerates other antioxidant compounds, including vitamin E, to exert antioxidant effects [179]. CoQ10 deficiency can contribute to the clinical manifestations of epilepsy [177]. Supporting this, one study found that patients with epilepsy had significantly lower CoQ10 levels than healthy controls [177]. In this study, decreased serum CoQ10 levels were correlated with more frequent seizures and a longer duration of epilepsy. CoQ10 has also shown promising effects when used in combination with traditional anti-epileptic drugs. In one study, CoQ10 and valproic acid reduced oxidative stress and prevented histopathological damage to the brain and liver more effectively than valproic acid alone [26]. This suggests that the administration of CoQ10 and valproic acid in combination can prevent the hepatotoxicity of valproic acid while potentiating its anti-epileptic activity [26]. Another study examined the efficacy of CoQ10 along with the ASM phenytoin in rats with pilocarpine-induced seizures. In this study, CoQ10 reduced the severity of seizures and alleviated oxidative stress [180]. Together, these studies suggest that CoQ10 can also be an effective and well-tolerated adjuvant therapy for epilepsy.

### 9.6. Astaxanthin

Astaxanthin is a carotenoid found in microalgae, yeast, and marine organisms, including salmon, shrimp, krill, and crayfish [181]. Astaxanthin can easily cross the BBB without causing toxicity [182]. This strong antioxidant decreases ROS generation and prevents oxidative damage [183,184,185]. Moreover, astaxanthin has anti-apoptotic, anti-inflammatory, and immune-enhancing activity [186,187,188]. In various neurological disorders, astaxanthin was found to mitigate brain damage and cognitive deficits [189]. A study of rats treated with astaxanthin starting shortly after SE onset found that treatment improved cognitive performance in a test of spatial memory [181]. Astaxanthin treatment reduced the inflammation observed in the brains of these rats, and this anti-inflammatory mechanism might be responsible for its neuroprotective effects [181].

## 10. Epilepsy and AEDs

More than two dozen AEDs are currently available for the treatment of epilepsy [190,191]. Pharmacologic strategies achieve seizure remission in an estimated 65–80% of patients with epilepsy [192,193]. AEDs can be used alone or in combination, although they are often used as monotherapy to prevent toxicity [194]. Classical AEDs such as valproic acid, levetiracetam, and benzodiazepines are frequently used as a first-line treatment against myoclonic seizures [195].

### 10.1. Valproic Acid

Valproic acid is widely used with considerable efficacy in treating simple and complex seizures during epilepsy. [196,197,198,199] It can be used as either monotherapy or polytherapy. In one study, valproic acid treatment in PTZ-treated mice exhibited neuroprotection, including reduced histopathological alterations, improved behavioral symptoms, increased antioxidant levels, and decreased inflammation, as evidenced by reduced TNF-α expression [183]. Furthermore, co-administration with astaxanthin offered greater benefits against epilepsy [183]. It is important to note that chronic valproic acid administration can increase ROS levels within cells, inducing the occurrence of seizures. Another risk of valproic acid is its reported hepatotoxicity, as evidenced by marked increases in serum levels of the aminotransferases AST, ALT, and ALP in rats treated with valproic acid [200]. Interestingly, co-administration of ellagic acid reduced valproic acid-induced hepatic injury in these rats [200].

### 10.2. Levetiracetam

Levetiracetam is a more recent AED that is effective in the control of partial-onset seizures [201,202,203,204,205]. Levetiracetam’s proposed mechanism of action is its ability to bind to synaptic vesicle protein 2A (SV2A), which prevents Ca^2+^ release from presynaptic neurons [206,207]. In this manner, levetiracetam can act as a neuromodulator. Compared to the characteristics of older AEDs, levetiracetam is thought to be more efficacious with lower toxicity [208,209]. In 1 study involving 145 people in a group receiving levetiracetam, it was found that SE resolved and functioning was enhanced in 47% of patients [210]. There was 1 meta-analysis on levetiracetam in children with focal seizures that found a 55% median reduction in seizure occurrence [194]. There was 1 group that conducted a randomized, double-blind study of 114 children and adults who had at least 12 seizures in the previous year despite pharmacological treatment [194]. The group that was provided levetiracetam as an adjunctive therapy had a 38.7% reduction in seizure frequency, compared to 14.3% in the group provided a placebo [194]. Notably, levetiracetam was effective in alleviating refractory epilepsy in both adults and children [194]. Similarly to other AEDs, levetiracetam might also be effective as one element of polytherapy. In PTZ-injected rats, the combination of levetiracetam and sodium selenite was more protective than levetiracetam monotherapy in delaying epilepsy progression and improving performance on behavioral tests [211].

## 11. Epilepsy and ASMs

The majority of currently available ASMs reduce neuronal excitability and seizure occurrence, although they might not treat the underlying etiology of epilepsy [212,213,214,215]. Many ASMs exert an anti-convulsive effect by repressing excitatory neurotransmission through their targeting of ion channels [216,217,218].

### 11.1. CBD

CBD is a cannabinoid without psychoactivity that has been investigated as an adjuvant for AEDs [219,220,221]. This is due to CBD’s anti-inflammatory properties, including its ability to prevent microglia activation and the release of inflammatory factors from astrocytes [222,223]. The efficacy of CBD in reducing seizure frequency has been demonstrated in both humans and animal models [224,225]. For instance, CBD has proven beneficial in clinical trials for medically refractory epilepsy syndromes [226]. A survey of 117 parents of children with epileptic spasms or Lennox–Gastaut syndrome found that 85% of participants felt CBD improved seizures, and 14% observed a complete absence of seizures when CBD was used [223]. Furthermore, a study analyzing 580 children and adults with drug-resistant epilepsy found that 12 weeks of CBD treatment reduced the median convulsive seizure frequency per month by 51% and total seizure frequency by 48% [223]. Additional evidence supporting the use of CBD in epilepsy comes from an open-label study of 162 patients with epilepsy originating in childhood. CBD treatment for 12 weeks reduced the monthly seizure frequency by an average of 36.5% [227].

CBD has also been shown to be effective as an adjunctive therapy alongside other ASMs. This is supported by both case studies and clinical trials. In one report, three pediatric patients with medically refractory epilepsy from Rasmussen encephalitis were provided adjunctive CBD along with their ASMs [228]. The inclusion of CBD offered clinical benefits beyond what would be expected from including an additional ASM in the treatment regimen [228]. Moreover, in four randomized clinical trials, CBD administered as an adjunctive therapy more effectively reduced seizure frequency than a placebo in patients with Lennox–Gastaut syndrome and Dravet syndrome [229].

### 11.2. Brivaracetam

Brivaracetam is a recently approved ASM that is being used as an adjunctive therapy for patients with focal seizures [230,231,232,233,234]. Brivaracetam has a similar mechanism of action as levetiracetam in that it exhibits high-affinity binding to SV2A vesicles. Additionally, it shows linear pharmacokinetics [235]. Some evidence indicates that brivaracetam is also effective in pediatric patients with focal seizures [236]. In 1 study analyzing 34 such patients aged 3–17 years, 16 patients responded significantly after 3 months of brivaracetam treatment. Ten of these patients had complete resolution of focal seizures [235]. A study of 200 adults with medically refractory epilepsy who were treated with brivaracetam found that 23% experienced at least a 50% reduction in seizure frequency [231]. Other research indicated that 50 mg/day of brivaracetam is an effective dose to significantly reduce seizure frequency [237]. This dose was also well tolerated, with rare adverse effects. Another use of brivaracetam and levetiracetam is SE treatment, allowing the two drugs to be used in emergency cases [238]. Although the two drugs have a similar mechanism of action, brivaracetam is suggested to be less likely than levetiracetam to cause adverse behavioral effects [239,240]. Therefore, some patients would benefit from switching from levetiracetam to brivaracetam [239,240].

### 11.3. Ursolic Acid

Ursolic acid has been demonstrated to prevent oxidative stress by inhibiting ROS generation [241,242,243,244]. It has also been shown to have anti-inflammatory effects, including inhibiting MAPK signaling to prevent NF-κB translocation and subsequent secretion of inflammatory compounds [245]. Through its attenuation of oxidation and inflammation, ursolic acid can exert a substantial neuroprotective effect [216,246]. In one study, these properties of ursolic acid allowed it to decrease seizure susceptibility and improve cognitive dysfunction in rats injected with pilocarpine [216]. During SE, GABAergic interneurons are often damaged or lost, which removes inhibitory signals by GABA from the neural circuitry [247]. Notably, ursolic acid has been observed to preserve GABA levels by inhibiting GABA transaminase [248]. Moreover, ursolic acid was found to prevent the loss of GABAergic interneurons in the previously described pilocarpine-induced rat model [216]. This suggests enhanced inhibitory neurotransmission as a possible mechanism by which UA dampens the cellular effects of SE.

### 11.4. Curcumin

Curcumin, which is produced by the herb *Curcuma longa*, possesses a broad range of activities, and it has been used as a traditional remedy for seizures [249,250,251,252]. The antioxidant properties of curcumin have been demonstrated in various epilepsy models, including KA, amygdala kindling, and post-kindled models [149,253,254,255]. Moreover, curcumin was found to prevent the spread of electrical activity to form generalized seizures in an iron-induced epilepsy model [256]. Similarly, *C. zedoaria* extracts were used as a treatment in rats kindled with PTZ injection [257]. *C. zedoaria* extract, which contains compounds including curcumin, elevated the tonic seizure threshold and decreased mortality [257]. Moreover, *C. zedoaria* extract improved performance in learning and memory among these rats, with one potential mechanism for this benefit being the extract’s enhancement of GABAergic signaling [257].

## 12. Epilepsy and Neuromodulation

Neuromodulation is a palliative treatment for patients with chronic drug-resistant seizures [40,258,259,260,261]. It encompasses the application of direct or induced electric currents to alter neural activity. Neuromodulation has been pursued as a strategy to reduce the occurrence and duration of seizures in patients with epilepsy who do not respond well to medication [262,263]. Neuromodulation consists of both invasive and non-invasive therapies. Invasive methods include VNS, deep brain stimulation, which uses implanted electrodes, and responsive neurostimulation, which is activated when a seizure is detected [264,265,266,267,268]. Less-invasive treatment options include transcutaneous VNS, transcranial direct current stimulation, and trigeminal nerve stimulation [269,270,271,272,273,274,275]. As a whole, neuromodulation strategies can induce a 30%–40% decrease in seizure occurrence after 3 months of treatment [258]. Only a small fraction of people maintain a total absence of seizures for at least 1 year after neuromodulation, but the majority have over a 50% decrease in the frequency of seizures [258].

### 12.1. VNS

VNS entails the use of a pulse generator to administer periodic electrical impulses to the vagus nerve [40,276,277]. This method can be especially beneficial in patients with medically refractory epilepsy who would also not be indicated for curative surgical treatment [278,279]. VNS achieved a greater than 50% reduction in seizure frequency in half of the patients, although fewer than 5% experienced total resolution of seizures [280,281]. VNS is effective even over a long period, and its ability to control seizures can improve over time [40]. The vagus nerve may inhibit the formation of seizures in more excitable regions of the brain, including the thalamus, thalamocortical projections, and limbic system [258]. This presents one mechanism of action for VNS in epilepsy. In addition, VNS increases serotonin and norepinephrine release through its activation of the raphe nuclei and locus coeruleus. Increased serotonin and norepinephrine transmission can be preventive against epilepsy [282,283].

### 12.2. Epilepsy and Surgery

Surgical interventions for epilepsy include curative procedures, palliative procedures such as corpus callosotomy, and implantation of devices for neuromodulation [284]. In its curative form, surgery can limit seizure spread and reduce seizure frequency by removing cortical areas that are necessary for the generation of seizures [41,285]. However, curative surgery prioritizes the preservation of normal cognitive abilities [40]. The ability of curative surgery to completely eliminate epilepsy is influenced by many variables, including epilepsy type, etiology, and the extent of resection [286]. Overall, surgery is a highly safe and efficacious option for treating epilepsy, although it has been underutilized [287,288]. Some evidence indicates that surgery can be more effective than medication for some patients with TLE [289]. In one study, patients with medically refractory TLE were randomized to either receive temporal lobe resection or continue drug therapy [290]. In total, 58% of patients who underwent surgery experienced complete elimination of seizures at a 1-year follow-up, compared to 8% of patients on AEDs [290]. Surgical removal of the sites of seizure origination may be a necessary strategy for patients with multidrug-resistant epilepsy [18]. For the third of patients with focal epilepsy who cannot find symptom control with medications, surgery offers an opportunity to alleviate or resolve seizures [284].

## 13. Epilepsy and Diet Therapy

The ketogenic diet consists of high fat content, sufficient protein levels, and extremely low carbohydrate intake [291,292,293,294]. It has classically been used as a dietary treatment for epilepsy [295,296,297,298]. Several trials described the efficacy of ketogenic diets in patients with pediatric epilepsy. A randomized controlled trial found that 38% of pediatric patients on a ketogenic diet had at least a 50% reduction in seizure frequency after 3 months, compared to only 6% of controls [299]. Furthermore, 7% of the ketogenic diet group had a near-total seizure reduction of at least 90%, which was not observed in any controls [193]. In another study evaluating 6 months of ketogenic diet consumption, the overall seizure frequency in pediatric patients was reduced by 70.79%, and the seizure severity was decreased by 35% [300]. The ketogenic diet is especially beneficial as a treatment option for medically refractory epilepsy when pharmacological strategies do not provide sufficient seizure control [301]. In trials of pediatric patients with drug-resistant epilepsy, the ketogenic diet can decrease the seizure frequency by more than 50% in up to half of the participants [302,303]. For instance, 1 study of 90 children <6 years old included controls, patients with refractory epilepsy treated with AEDs, and patients with refractory epilepsy on a ketogenic diet [301]. Compared to the group on AEDs, the group on a ketogenic diet had a lower seizure frequency and severity, as well as higher total antioxidant capacity [301].

Consistent with this finding, the ketogenic diet is believed to function in part through its antioxidant mechanism [304]. It can increase the GSH availability within cells and protect mtDNA from oxidative damage while reducing ROS formation within mitochondria [3,98,305]. The ketogenic diet can result in the formation of ketone bodies, which can generate acetyl-CoA for ATP synthesis and reduce ROS generation [306,307]. This also prevents the opening of the MPTP and subsequent release of excess Ca^2+^ [308,309]. These activities of the ketogenic diet contribute to its protective effect against oxidative stress. The ketogenic diet has additionally been demonstrated to have anti-inflammatory activity in an animal model of spinal cord injury [310]. The ketogenic diet may modulate neuroinflammatory pathways that cause seizure-induced neuronal loss [311,312]. The ketogenic diet might also enhance GABA production and inhibit glutamate synthesis, thereby exerting effects against epilepsy [313]. This is corroborated by evidence that patients on a ketogenic diet have higher cerebrospinal fluid levels of GABA [306].

## 14. Epilepsy and Nutrients

Nutrients with known antioxidant or anti-inflammatory activity include vitamin A, vitamin C, omega-3 fatty acids, polyphenols, and carotenoids [314,315,316,317,318,319]. Vitamins can offer benefits against epilepsy, especially when used as an adjunctive therapy [320]. Multivitamin therapy, including vitamin B6, vitamin B9, vitamin D, vitamin E, and CoQ10, administered adjunctively, reduced the average monthly seizure frequency from nine to two [321]. After 6 months of treatment, 63% of individuals had at least a 50% decrease in seizure occurrence [321]. Although vitamin B6 has specifically been demonstrated to lead to better outcomes in epilepsy, it is important to note that it does not provide benefits for all patients [322,323,324,325].

Vitamin D supplementation is also promising for epilepsy treatment, particularly because several investigations have observed vitamin D deficiencies among patients with epilepsy [326,327,328,329]. In 1 clinical trial, a treatment arm investigated 4 weeks of treatment with 4000 IU/day of vitamin D3, followed by 4 weeks of treatment with 16,000 IU/day of vitamin D3 [213]. This treatment group had nearly a 70% decrease in the average seizure frequency [213]. Among individuals with medically refractory epilepsy and vitamin D3 deficiency, administering vitamin D3 was found to reduce seizure frequency by up to 40% [330]. In a pediatric study of 648 children with epilepsy, vitamin D supplementation also led to more effective seizure control [329]. The findings from clinical trials were corroborated by animal models of epilepsy, in which vitamin D administration had anti-seizure activity and vitamin D receptor knockout mice had more frequent seizures [331].

A study in which 400 IU of vitamin E were administered to patients with epilepsy for 3 months recorded a nearly 60% reduction in seizure frequency [323]. Vitamin E has been especially promising as long-term adjunctive therapy in refractory epilepsy [332,333]. As an antioxidant, vitamin E promotes the clearance of ROS and prevents oxidative damage to proteins and lipids [334,335]. In rats with pilocarpine-induced seizure, vitamin E provided neuroprotection, evidenced by its ability to increase CAT levels and mitigate the increase in free fatty acid levels in the brain [336].

Another vitamin that could offer benefits for patients with epilepsy is vitamin C, especially because this group has been observed to have lower serum vitamin C levels [337]. In several models of epilepsy, including pilocarpine-, PTZ-, and penicillin-induced epilepsy, vitamin C improved seizure control and outcomes, such as mortality and seizure latency [338]. Studies of animal models of epilepsy revealed that vitamin C could mitigate oxidative stress, which might explain its ability to control seizures [339,340].

### 14.1. Fish Oil and Fatty Acids

Another nutrient that has reduced seizure frequency among patients with epilepsy is fish oil [341,342,343]. Because fish oil is established to be safe within a dose of 4 g/day, its administration as an adjunctive supplement could offer benefits in managing epilepsy with little risk of adverse effects [344]. There was 1 study that found that 0.6–2 g/day of fish oil decreased seizure frequency and duration. Conversely, some trials did not find an effect of fish oil and omega-3 polyunsaturated fatty acids on seizure suppression [345,346,347]. Short-chain fatty acids are also promising as part of an epilepsy treatment regimen because they have antioxidant and anti-inflammatory effects [348].

### 14.2. Magnesium and Zinc

Patients with epilepsy display reduced levels of magnesium, which has been proposed to be a cause of seizures [349,350,351]. Consistent with this, the severity of epilepsy is correlated with the degree of magnesium deficiency [352]. Zinc supplementation might also be beneficial for epilepsy, as evidenced by the administration of zinc in a PTZ-induced rat model of epilepsy. Zinc was found to mitigate epileptogenesis, prevent oxidative stress, and reduce neuroinflammation [353].

### 14.3. Polyphenols and Flavonoids

Polyphenols are compounds that can cross the BBB and serve as neuromodulators. Therefore, they are being considered for their potential to ameliorate CNS diseases [136,354,355]. In particular, polyphenols might be able to disrupt the course of epileptogenesis that gives rise to recurrent seizures [38]. For instance, the polyphenol resveratrol was found to prevent neurodegeneration in a KA model of SE and reduce oxidative stress and neuroinflammation [356]. Another study of a KA rat model of epilepsy found that 10-day resveratrol (15 mg/kg once daily) treatment prevented neuronal loss and decreased the frequency of seizures [357].

Flavonoids, as a class, are promising nutritional treatments for epilepsy and CNS disorders because of their antioxidant properties [136]. Additionally, flavonoids can increase the activity of GABA receptors, increasing the strength of inhibitory neurotransmission [136]. Some evidence suggests that the flavonoid quercetin can improve outcomes in epilepsy, especially because it was found to reduce inflammation in KA-induced epilepsy models [358]. As part of its anti-inflammatory effect, quercetin blocks microglial activation and pro-inflammatory cytokine secretion. Quercetin might especially be helpful as an adjunctive therapy because when administered with levetiracetam, it alleviated depression that was comorbid with epilepsy [358].

## 15. Conclusions

Epilepsy is a CNS disorder with a high prevalence that carries a significant burden through the presence of recurring seizures. Because the brain has the highest demand for oxygen consumption among all organs, it is especially vulnerable to oxidative stress and subsequent damage. ROS generation and oxidative stress can contribute to epileptogenesis and eventual neuronal death. In addition, oxidative stress can increase neuronal hyperexcitability and increase the likelihood of seizure occurrence. Another process contributing to epilepsy pathophysiology is mitochondrial dysfunction, which can induce neuronal death, a feature that has also been observed in epilepsy. Furthermore, neuroinflammation is proposed to be a key contributor to the onset and progression of epileptic seizures. In addition, seizures can contribute to processes such as oxidative stress and inflammation, leading to the progression of epilepsy.

Various therapeutic strategies are available to treat epilepsy, including AEDs, ASMs, and antioxidants. By preventing the accumulation of ROS and free radicals and guarding against oxidative stress, antioxidants can address one aspect of epilepsy pathophysiology. Most ASMs that are presently available seek to target seizures rather than epilepsy pathophysiology, and they work by decreasing neuronal excitability. Although many medications are available to control epilepsy, approximately one-third of patients continue to have seizures that cannot be resolved with medication. These patients with medically refractory epilepsy can have a lower quality of life, cognitive deficits, and low mood. In these cases, other treatment options include surgery, neuromodulation, and dietary strategies. Understanding the consequences of diet therapies such as the ketogenic diet and specific nutritional supplements such as antioxidant vitamins can support the further development of nutritional strategies in epilepsy treatment. Targeting processes underlying epileptogenesis, such as oxidative stress, inflammation, and mitochondrial dysfunction, may be a fruitful area of investigation for new antiepileptic therapies. Increasing the range of available interventions may provide alternative treatment options for medically refractory epilepsy. This review provides an overview of several causative processes in epilepsy and how they correspond to specific treatment strategies. Through a discussion of epilepsy pathogenesis and promising therapeutic strategies, this review can provide insight into avenues for the future development of clinical interventions for epilepsy.
